# The Role of Oxidative Stress and Antioxidants in Diabetic Wound Healing

**DOI:** 10.1155/2021/8852759

**Published:** 2021-02-04

**Authors:** Liling Deng, Chenzhen Du, Peiyang Song, Tianyi Chen, Shunli Rui, David G. Armstrong, Wuquan Deng

**Affiliations:** ^1^Department of Endocrinology, Multidisciplinary Diabetic Foot Medical Center, Chongqing Emergency Medical Center, Chongqing University Central Hospital, Chongqing 400014, China; ^2^Department of Surgery, Keck School of Medicine of the University of Southern California, CA, USA

## Abstract

Foot ulcers are one of the most common and severe complication of diabetes mellitus with significant resultant morbidity and mortality. Multiple factors impair wound healing include skin injury, diabetic neuropathy, ischemia, infection, inadequate glycemic control, poor nutritional status, and severe morbidity. It is currently believed that oxidative stress plays a vital role in diabetic wound healing. An imbalance of free radicals and antioxidants in the body results in overproduction of reactive oxygen species which lead to cell, tissue damage, and delayed wound healing. Therefore, decreasing ROS levels through antioxidative systems may reduce oxidative stress-induced damage to improve healing. In this context, we provide an update on the role of oxidative stress and antioxidants in diabetic wound healing through following four perspectives. We then discuss several therapeutic strategies especially dietary bioactive compounds by targeting oxidative stress to improve wounds healing.

## 1. Introduction

Diabetes mellitus (DM) and its complications are increasingly prevalent worldwide with a serious burden on patients and health care systems [1] Diabetic foot ulcers have a substantial impact on disability, morbidity, and mortality. The mechanism of diabetic wound chronicity has not been well understood. It is currently believed that oxidative stress plays a vital role in the occurrence and development of diabetic wound [2, 3]. Oxidative stress is caused by overproduction of reactive oxygen species and insufficient antioxidant systems. However, the process of oxidative stress in wound development and healing remains unclear. This review will further develop the discussion on how oxidative stress may affect diabetic wound healing in terms of skin injury, neuropathy, arterial disease, and infection. Furthermore, the plausible role of antioxidants including plant bioactive compounds on promoting wound healing will be addressed in order to explore novel approaches and strategies for promotion of diabetic wound healing.

## 2. Oxidative Stress State in the Development and Healing of Diabetic Wound

**Figure 1:**
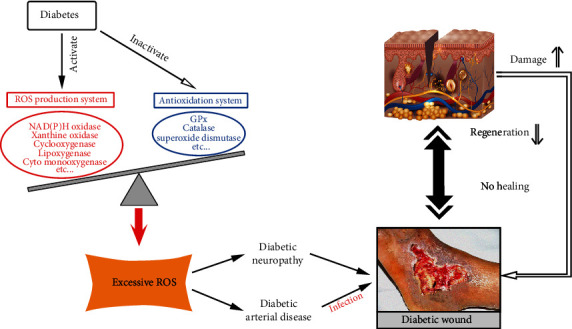
The role of oxidative stress in development and chronicity of diabetic wound.

Reactive oxygen species (ROS) are crucial regulators of several phases of wound healing. Indeed, low levels of ROS are required for the fight against external damage [4]. However, excessive oxidative stress on tissues and the decrease of antioxidant ability results in redox imbalance, which is a major cause of nonhealing diabetic wounds [5]. Clinical studies investigated that nonhealing diabetic wounds are infiltrated by the highly oxidizing environment, which is associated with hyperglycemia and tissue hypoxia, that leads to delayed wound repair. People with long-term type 2 diabetes have significant reductions in the antioxidant enzyme activity [6]. Oxidative stress may influence diabetic wound healing through skin injury, neuropathy, ischemic lesion, and topical infection (Figure 1).

### 2.1. Diabetic Skin Injury

The repair process of diabetic skin injury includes the temporally overlapping stages of coagulation, inflammation, migration-proliferation, and remodeling [7].While the molecular mechanisms underlying such defects have not yet been clarified, some have reported that a hyperglycemic state directly affects the keratinocyte and fibroblast activity inducing changes in protein synthesis, proliferation, and migration, reduced antimicrobial peptide expression, and increased oxidative stress. These myriad changes may result in the injury of the skin barrier function making the skin susceptible to damage and infection [8–11]. A balanced redox state is likely critical for prompt healing. Diabetic skin appears to be infiltrated with more inflammatory cells, edema, and less granulation tissue formation than the normal skin, suggesting a deterioration in diabetic ulcer healing reserve [2, 7].Additionally, the periwound and central wound tissue in people with diabetes appears to also be more likely to be prone to excessive oxidative stress and damage, which results in poor healing [12, 13]. Kim et al. suggested that both high oxidative stress levels and bacteria set the wound up for chronic wound development [14].

Experiments that have evaluated hypoxia and oxidative stress suggest that they may lead to the downregulation of miRNA biogenesis genes in cultured fibroblasts [15]. Recent efforts have indicated the levels of serum advanced glycation end product (AGE), and its epidermal receptors were elevated in diabetes [9, 16]. AGEs bind to receptors on the endothelial cell surface inducing the overproduction of ROS [17]. AGE in the diabetic wound microenvironment appears to impair wound contraction and prolongs the inflammatory response and appears to deleteriously damage extracellular matrix (ECM) proliferation [18]. Nrf2 is a key transcription factor involved in wound healing by regulating angiogenesis and antioxidant gene expression, which is impaired in diabetes [19, 20]. A recent study indicates that activation of Nrf2 signaling significantly increased TGF-*β*1 and reduced MMP9 in keratinocytes in diabetic wound healing [12]. Another study reported that SIRT1 (silent mating information regulation 2 homolog) activation could protect vascular endothelial cells from oxidative stress damage to improve angiogenesis in wounds to accelerate wound healing in diabetic mice, and this effect may be through the AKT-Nrf2 pathway [21]. Sirtuins are class III histone deacetylase enzymes which are evolutionarily conserved and possess NAD + dependent deacetylase activity. Sirtuin 3 (SIRT3) is a protein deacetylase located in the mitochondria. It serves to regulate the mitochondrial function, which is involved in regulating cellular redox status, mitochondrial energetics, biogenesis, dynamics, and apoptosis. Recent work has demonstrated that the lack of SIRT3 reduced blood supply, inhibited vascular endothelial growth factor (VEGF) expression, promoted superoxide production, impaired total antioxidant capacity, and consequently resulted in delayed skin wound healing in diabetic mice [22]. Therefore, sirtuins may be a new therapeutic target to improve diabetic wound healing.

### 2.2. Diabetic Neuropathy

At least half of the people with diabetes will develop clinically significant peripheral neuropathy [23–25]. Sensory nerve dysfunction leads to the loss or weakening of skin protection. Motor neuropathy, which increases plantar pressure that directly destroys the tissue, causes plantar capillary occlusion, local tissue ischemia, and destruction [26]. The autonomic neuropathy of sweat glands in people with diabetes leads to reduced skin sweating, abnormal temperature regulation, and dry and chapped skin, which in turn damages the integrity of the skin, leading to a reduced barrier to infection. It also leads to perturbations in the skin blood flow and microcirculatory disorders such as loss of peripheral sympathetic nerve innervation and tension, leading to vasomotor dysfunction and abnormal arteriovenous shunting. As a result, the abnormal blood flow distribution and nutritional capillary ischemia could occur.

**Figure 2:**
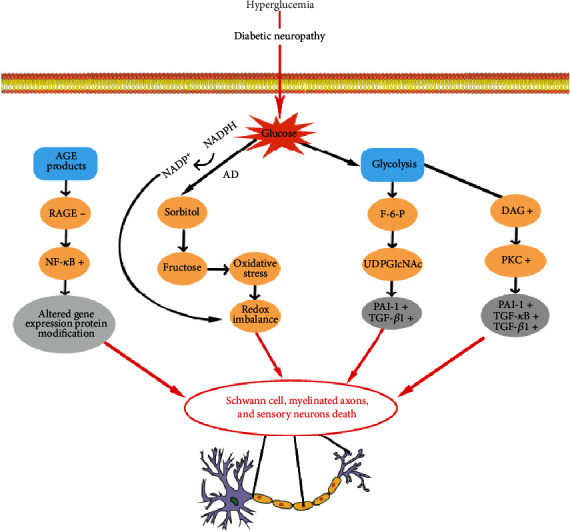
The main pathways with oxidative stress in diabetic neuropathy.

It has been well demonstrated that hyperglycemia is a critical mechanism in the induction of oxidative stress. Increased oxidative stress appears to deleteriously affect blood supply, structure, and metabolism of the peripheral nerve [26] that leads to extensive deterioration to the all aspects of the peripheral nervous system including Schwann cells, myelinated axons, and sensory neurons located in the dorsal root ganglia [27]. Meanwhile, insufficient ATP supplies could lose the ability to transport axons, as axons were rich in mitochondria, providing direct nerve energy supply, thereby further promoting axonal injury, causing diabetic neuropathy. The loss of ability of detoxify the excess oxidative stress with insufficient ATP supplies led the axons oxidative stress damage in hyperglycemia, which precipitated axonal degeneration or apoptosis [28]. Studies have suggested that multiple biochemical pathways are deleteriously affected by oxidative stress (Figure 2).

#### 2.2.1. Activated Polyol Pathway

The polyol pathway mainly includes the conversion of glucose to sorbitol by aldose reductase (AR) and the conversion of sorbitol to fructose by sorbitol dehydrogenase. In patients with diabetes, elevated intracellular levels of glucose cause the affinity of AR for glucose to also increase. This then leads to the increased production of sorbitol. Subsequent accumulation of sorbitol reduces the activity of Na+K+-ATPase which reduces the physiological reserve of nerve cells and commensurate reduction of nerve conduction velocity. It is also understood that the hyperglycemia-induced polyol pathway leads to increased oxidative stress because of depletion of NADPH, which is derived from the pentose phosphate pathway that generates GSH from glutathione. Meanwhile, excess fructose, as a product in metabolism, accelerates glycation and further consumption of NADPH to aggravate intracellular oxidative stress [29]. It has been confirmed in diabetic mice with high expression of aldose reductase. Diabetic mouse models indicate that sorbitol accumulates significantly in the sciatic nerve of diabetic AR+/+ mice. Traces of oxidative stress and DNA damage in the sciatic nerve were additionally observed [30]. Until now, aldose reductase inhibitors have been used to inhibit oxidative stress through the polyol pathway [31]. This may be beneficial for improving diabetic ulcer with concomitant neuropathy.

#### 2.2.2. Activated Hexosamine Pathway

In an environment characterized by hyperglycemia, fructose-6 phosphate, a metabolic intermediate of glycolysis, is shifted from the glycolytic pathway to the hexosamine pathway and then converted to uridine diphosphate N-acetylglucosamine (UDPGlcNAc). Subsequently, UDPGlcNAc attaches to the serine and threonine residues of transcription factors [32].

Studies have reported activation of the hexosamine pathway leading to the impaired nerve function. In addition, the hexosamine pathway can promote the expression of cytokines such as TGF-*α* and TGF-*β*1 [33, 34].

Hyperglycemia activates the hexosamine pathway that eventually results in increasing of transcription factor Sp1, which is promoting the overexpression of TGF-*β*1 and plasminogen activator inhibitor-1(PAI-1) in arterial endothelial cells [35, 36]. In addition, hyperglycemia induces the hexosamine pathway, and NF-*κ*B signaling increases the expression of thrombospondin 2 (TSP2). TSP2 is a critical matricellular protein in injury responses. This appears to delay diabetic wound healing [37].

#### 2.2.3. Activated AGE Pathway

In DM, nonenzymatic glycosylation of glucose can chemically interact with amino acids, lipids, and nucleic acids, which lead to the formation of reversible early glycosylation products. After chemical reconstitution irreversible AGEs subsequently form, AGE receptors exist in a variety of cell types such as vascular endothelial, vascular smooth cells, and others. As AGEs combine with their receptors, ultimately, extracellular and intracellular structure and function are altered [38].

The formation of AGEs on ECM molecules can increase ECM production and tissue stiffness directly or indirectly through activation of TGF-*β* receptors to stimulate cell growth. A signaling cascade triggered by the binding of AGEs to its receptor on the endothelial cell surface leads to activation of NADPH oxidase and overproduction of ROS, p21, RAS, and MAPKs. NF-*κ*B is a key target of AGE-RAGE signaling. Excess oxidative stress derived from the AGE-RAGE axis activates NF-*κ*B and affects transcriptional activation of numerous cytokines and adhesion molecules including endothelin-1, ICAM-1, E-selectin, and tissue factor, many of which are closely linked to inflammation and diabetic angiopathy [17].

AGEs also reduce the bioavailability of endothelium NO and cause the overproduction of ROS [39, 40]. It has been suggested that the AGEs-RAGE axis contributes to microvascular and macrovascular complications of diabetes [41].Thus, the process of the AGE pathway might induce neuropathy via impairing vasodilation, thickening capillary basement membrane, and endothelial hyperplasia.

In addition to the aforementioned effects, AGEs negatively impact diabetic ulcer healing by increasing apoptosis, decreasing the proliferation of fibroblasts, and reducing the activity of growth factors such as fibroblast growth factor [42]. Moreover, AGE-induced autophagy is associated with delayed cutaneous ulcer healing through stimulation of M1 polarized macrophage [43, 44].

#### 2.2.4. Activated PKC Pathway

PKC is comprised of a group of serine/threonine protein kinases with important physiological functions affecting many cellular signaling transduction pathways [45]. There are several isoforms of PKC, which play a critical role in multiple biological systems. PKC*α*, *β*I, *β*II, and *δ* are main components of the peripheral nerve [46]. In the environment of hyperglycemia, overproduction of oxygen free radicals inhibits the activity of glyceraldehyde phosphate dehydrogenase (GAPD). This leads to increased conversion of dihydroxyacetone phosphate (DHAP) to diacylglycerol (DAG), which in turn activates the PKC pathway [47]. Activated PKC can mediate the production of oxygen free radicals, inhibiting the activity of NO, thus leading to damage of the endothelial function.

A diabetic mouse study paradoxically reported decreased PKC activity. The difference from the previously stated results was likely due to the reduced expression of membrane PKC-*α* translocated to the cytoplasm, while the expression of membrane PKC*β*II increased [48]. Recent studies have reported improved neural function of diabetic rats with the administration of nonselective PKC isoform inhibitors or selective PKC *β* inhibitors. In addition, the sustained increasing PKC*δ* of human fibroblasts of DM hindered wound healing and insulin signaling was observed, thereby PKC*δ* inhibition may be a novel approach for treating diabetic wounds [49]. In summary, the mechanism of the PKC pathway in diabetic complications remains unclear.

Recent experiments have suggested that benfotiamine, a lipid-soluble analogue of vitamin B1, might improve diabetic wound healing via preventing the activation of the hexosamine pathway and the AGE and PKC pathway [50]. Preclinical studies showed that stimulating Nrf2-mediated antioxidant in the local regenerative environment of diabetic wounds significantly improved the molecular and cellular composition of wound beds [51].

The process of OS is irreversible and exerts a memory effect on metabolism with the correction of hyperglycemia [52, 53]. It was well demonstrated that the duration and severity of hyperglycemia were associated with the occurrence and progression of diabetic neuropathy, and enhanced glucose control delayed the process of developing clinical neuropathy in type 1 diabetes mellitus [3, 54]. However, conclusions in type 2 diabetes mellitus remain elusive [3, 55]. Indeed, strict glucose control does appear to impair nerve conduction velocity and vibration threshold in type 2 diabetes mellitus. This might be because neurons are more prone to severe hypoglycemic episodes. With acute glucose fluctuations, the PKC pathway is activated while inflammatory factors and adhesion molecules were secreted [56, 57], which further results in endothelial dysfunction and oxidative stress of the peripheral nerve.

### 2.3. Diabetic Peripheral Artery Disease

The role of diabetic angiopathy is well described in diabetic wounds. Diabetic microangiopathy and macroangiopathy are the combined result of irreversible complex nonenzymatic glycation, elevated oxidative stress and inflammation, and endothelial dysfunction and hypercoagulability state [58].

Oxidative stress caused by activated biochemical pathways, such as the AGE/RAGE pathway, the polyol pathway, PKC activation, and the hexosamine pathway, results in production of inflammatory mediators, pericyte degeneration, thickening basement membrane, endothelial hyperplasia, NO reduction, impaired vasodilation, and increasing procoagulant biomarkers, such as IL-6, TNF-*α*, D-dimer, and PAI-1. All of these are in one way or another involved in the development of diabetic microangiopathy [59–62]. The alteration of the capillary or arteriolar vessel structure prevented the delivery of nutrients and activated white blood cells to specific tissues, increases the susceptibility and severity of infections, and accelerates the occurrence and progression of diabetic ulcer. In addition, microthromboembolism might be more prone to occur in terminal vessels of smaller diameter, which aggravates ischemia and hypoxia of the local tissue in diabetic wounds. SKQ1 as a mitochondria-targeted antioxidant increased the number of myofibroblasts, lowered the levels of neutrophils, and elevated macrophage infiltration; moreover, SKQ1 decreased lipid peroxidation levels without alteration of circulatory IL-6 and TNF levels. SKQ1 treatment also improved cell migration, thereby improving dermal wound healing of genetically diabetic mice [63].

The biochemical pathways participated in the development of diabetic macroangiopathy were as follows: AGEs-RAGE axis, polyol pathway, hexosamine pathway, PKC activation, overproduction of ROS, and chronic inflammation [64, 65]. In turn, atherosclerosis induced endothelial dysfunction and inflammation, and these processes are worsened in diabetes [59]. Moreover, recent evidences investigated that hypoglycemia may also play an vital role in vascular complications of diabetes [66]. Hyperglycemia or short-term glucose fluctuations induced OS and proatherogenic gene expression alteration which will be long-lasting later even in normal glycemic conditions [67]. As a result, the lumen of large vessels in diabetes narrow or abstract, and tissue ischemia deteriorated, which lead to a delay in diabetic wound closure.

### 2.4. Topical Infection

Patients with uncontrolled DM are more prone to skin infections such as fungal and bacterial infections. In turn, infections make the wound difficult to heal. The microbiome in diabetic wounds is closely related to the skin microbiome showing complex time-dependent features as well as individual differences. Diabetic wounds display disease-related changes. Staphylococcal species dominate [68–71].

## 3. Antioxidants in Diabetic Wound Healing

### 3.1. Antioxidant Effects of Antimetabolism Imbalance Drugs on Wound Healing

Metformin is a classic therapy for DM. Recent literatures have reported that metformin improved angiogenic functions of endothelial and endothelial progenitor cells by activating the NOS pathway, and it is effective in the treatment of skin wounds in diabetic mice [72, 73].

SGLT2 inhibitors, as a novel glucose-lowering approach, grow evidence suggesting that it can lower the mRNA expression of TNF-*α*, IL-6 and MCP-1, and infiltration of inflammatory cells in the plaque and adipose tissue to improve inflammation and endothelial function, which seem to be involved in the alleviation of atherosclerosis by empagliflozin [74, 75]. Furthermore, with SGLT2 inhibitor therapy, the proinflammatory phenotype and glucotoxicity in experimental diabetic animals were reversed [75]. In addition, hyperglycemia-induced mitochondrial dysfunction was relieved by SGLT2i in hyperglycemic mouse aorta vascular cultures [76]. Therefore, SGLT2i may be beneficial to diabetic wounds for healing.

Dipeptidyl peptidase-4 (DPP-4) inhibitors can lower blood glucose and reduce cardiovascular risk in patients with type 2 diabetes mellitus. Previous studies indicated that DPP-4 exerts a preventive effect on oxidative stress via Nrf2 or other pathways. Therefore, DDP-4 may be a treatment option for patients with diabetic chronic wounds [77, 78].

Fenofibrates, along with a proper diet to fight against high cholesterol and triglyceride levels, has been indicated to simulate keratinocyte differentiation and improve the skin barrier in vivo [79]. It also ameliorated oxidative stress and blockade of Wnt/*β*-catenin signaling [80]. These findings provide insights into treatment for diabetic ulcers. Systemically, use of statins exerts a reduction on cholesterol and can stimulate angiogenesis. Recent researches have revealed that stains can improve wound healing process in diabetic mice [81–83]. These may be a considerable therapy for patients with diabetic chronic wounds in clinic.

### 3.2. Antioxidant Effects of Drugs for Diabetic Neuropathy on Wound Healing

*α*-Lipoic acids (ALA) have been demonstrated to inhibit the progression of diabetic neuropathy by scavenging ROS, regeneration of endogenous and exogenous antioxidants, renovation of oxidized proteins, inhibition of NF-Kb, and regulation of gene transcription. Nowadays, it is widely used in the clinic for diabetic microangiopathy [64, 84, 85].

Coenzyme Q10, an endogenous synthesized lipid and a vitamin-like substance primarily present in the mitochondria, ameliorates oxidant stress and apoptosis [86]. CoQ10 is an electron transport in the mitochondrial respiratory chain, consequently increasing energy generation of ATP and enhancing the mitochondrial antioxidant activity [87]. Aldose reductase inhibitors are used to inhibit oxidative stress by the polyol pathway [31], and it may be beneficial for improving diabetic ulcer with neuropathy.

### 3.3. Antioxidant Effects of Drugs for Diabetic Angiopathies on Wound Healing

Cilostazol, a phosphodiesterase type 3 inhibitor, has been suggested to be involved in the procedure of antiatherosclerosis with antiplatelet and vasodilatory effects. A study on diabetic mice indicated that cilostazol significantly reduced RAGE, ROS, NF-*κ*B signaling, downstream gene expressions, and cell functions induced by hyperglycemia in VSMCs [88]. Low molecular-weight heparin ameliorates chronic diabetic wound healing, possibly by increasing microcirculation of wound margin [89, 90], but it is still controversy [91]. Iloprost improved limb perfusion and may be an important therapy for diabetic ulcerative lesions with severe ischemia [92]. However, there are only sparse data and lack of high-quality randomized controlled studies to show efficacy in any of these three agents [93].

### 3.4. Antioxidant Effects of Drugs for Diabetic Skin on Wound Healing

The epidermal growth factor (EGF) administration was beneficial for the alleviation of oxidative stress to improve wound repair [94]. Biogenic AgNPs synthesized from Brevibacillus brevis KN8 could inhibit the overexpression of MMP-2 and MMP-9 in granulation tissues and accelerated wound healing in diabetic mice beyond the antimicrobial activity [95]. Sodium pentaborate pentahydrate (NaB) displayed great antimicrobial properties and improved proliferation, migration, and the expression of growth factor and gene expression in dermis, and studies on diabetic rats proved that NaB improve diabetic wound healing rate [96].

### 3.5. Antioxidant Effects of Drugs for Diabetic Infection on Wound Healing

Strict antimicrobial dressing should be performed in diabetic ulcers with infection [97]. The microbiome in wound is associated to the skin microbiome showing complex and time-dependent features, as well as individual differences [68]. The microbial culture can be used to guide antimicrobial therapy.

Efficient antimicrobial treatment with daptomycin [98] exerted positive effects on wound healing at the molecular level, such as reducing the level of local IL-6 and MMP-9 and increasing TIMP-1 in the MRSA-infected diabetic wound.

### 3.6. Antioxidant Effects of Plant Ingredients on Wound Healing

Ferulic acid (FA) is a natural antioxidant derived from fruits and vegetables that inhibited lipid peroxidation and increased the expression of catalase, superoxide dismutase, glutathione, nitric oxide, and serum zinc and copper, probably improving the healing process in diabetic ulcer [99]. Besides, syringic acid treatment also promotes migration and proliferation to improve wound healing [100].

The fusion protein decreased serum proinflammatory cytokines such as IL-6, TNF-*α*, expression of cyclooxygenase-2, and increased activities of antioxidant enzyme including superoxide dismutase, glutathione peroxidase, and catalase, and it also increased proangiogenic cytokines levels including VEGF, intercellular adhesion molecule, and expression of VEGF, FGF-2, p-ERK, and p-Akt protein in granulation in diabetic rats, which significantly accelerated the diabetic wound healing [101].

Animal study has indicated chlorogenic acid, a dietary antioxidant, that could accelerate wound healing, enhance hydroxyproline content, decrease malondialdehyde/nitric oxide, and elevate the level of reduced glutathione in wound bed [102]. However, a number of researches are needed to furtherly confirm these data.

### 3.7. Antioxidant Effects of Potential Antioxidants on Wound Healing

The drug-loaded ROS-scavenging hydrogel promotes wound closure by decreasing the ROS level and upregulating M2 phenotype macrophages around the wound. Moreover, such hydrogels formed in wound also increased the release of granulocyte macrophage colony-stimulating factor to fight against external bacteria and improve the wound closure [103].

The experiment has indicated that deferoxamine (DFO) could reduce oxidative stress and promote hypoxia-inducible factor-1 alpha activation to facilitate neovascularization and regeneration in chronic diabetic wounds. Dominik Duscher has demonstrated the treatment of DFO and the enhanced concept drug delivery system treatment eTDDS that markedly increased wound vascularity, dermal thickness, collagen deposition, and tensile strength, thereby accelerating wound healing. Therefore, DFO eTDDS may have the potential in treatment of diabetic wounds [104].

In addition, vitamins A, C, and E are antioxidants [105]; however, some literature has reported that high doses of vitamins possibly increase the mortality of cardiovascular events and risk of tumors such as lung and skin tumors [106, 107]. Propolis could increase the glutathione (GSH) and GSH/glutathione disulfide (GSSG) ratio, deplete TNF, and increased interleukin10 levels to reduce the wound area [108]. Antioxidant with *N*-acetyl cysteine (NAC) is known to improve endothelial cell function and angiogenesis, and Mohamed et.al reported that daily NAC use improved postamputation stump healing, perfusion, neovascularization, and reduced muscle fiber damage [109, 110] (Table 1).

Further research is needed to confirm or refute whether these therapies are beneficial to diabetic wound healing. In addition, melatonin, taurine, and acetyl L-carnitine have been reported to improve oxidative stress in diabetic wounds in the recent literatures. However, a number of randomized controlled studies are needed to confirm.

## 4. Conclusion

Wound healing is a complex, dynamic process. However, most diabetic wounds are difficult to heal. Multiple factors such as hyperglycemia, neuropathy, blood supply, matrix turnover, wound contraction, and the microbiome play a role in the complex symphony of diabetic wound healing. Oxidative stress regulates wound healing through several related signal pathways. It therefore stands to reason that active control of ROS levels through antioxidants and antioxidative enzyme systems may reduce oxidative stress-induced damage to improve wound healing. Traditional systemic and topical medications could be beneficial for wound healing. Clinicians and scientists would do well to focus on development and testing of antioxidants to facilitate diabetic wound healing, thereby reducing the unnecessary burden of amputation, morbidity, and mortality in this population.

## Figures and Tables

**Table 1:** Antioxidants and their potential benefit in diabetic wound healing.

	Drugs	Observation	Mechanism	Reference
Antimetabolism imbalance	Metformin	Improved angiogenic functions of ECs and EPCs	NO, AMPK/mTOR pathway	[72, 73]
	SGLT2i	Improve inflammation and endothelial function	Lower mRNA expression of TNF-*α*, IL-6, and MCP-1	[74–76]
	DDP-4i	Antioxidative stress	Nrf2 pathway	[77, 78]
	Fenofibrates	Improve skin barrier	Wnt/*β*-catenin pathway	[79, 80]
	Statins	Promote angiogenesis and lymphangiogenesis	PI3-kinase/Akt pathway	[81–83]

Therapy for diabetic neuropathy	ALA	Antioxidative stress	Inhibition of NF-*κ*B	[64, 84, 85]
	Aldose reductase inhibitors	Antioxidative stress	Polyol pathway	[31]
	Coenzyme Q10	Ameliorates oxidant stress and apoptosis	Increasing energy generation of ATP	[86, 87]

Therapy for diabetic angiopathies	Cilostazol	Antiatherosclerosis		[88]
	Low molecular-weight heparin	Increase microcirculation	Anticoagulation	[89–91]
	Iloprost	Improve limb perfusion	/	[92]

Therapy for the diabetic skin	EGF	Antioxidative stress	/	[94]
	Biogenic AgNPs	Accelerate wound healing	Inhibit overexpression of MMP-2 and MMP-9, antimicrobial	[95]
	NaB	Accelerate wound healing	Antimicrobial activity and improve proliferation	[96]

Therapy for infection	Antibiotics	Fight against for infection	/	[97, 98]

Plant ingredients for OS	Ferulic acid	Antioxidative stress	Reduce MMP (2, 8, 9) and increase TIMP-1 and 2	[99, 100]
	Chlorogenic acid	Antioxidative stress	Elevate the level of reduced glutathione	[102]

Potential antioxidants	The drug-loaded ROS-scavenging hydrogel	Accelerate wound healing	Decrease ROS level and upregulate M2 phenotype macrophages	[103]
	Deferoxamine	Promote neovascularization and regeneration	Suppress OS and activate hypoxia-inducible factor-1 alpha ion	[104]
	Propolis	/	Increase GSH/GSSG) ratio	[108]
	*N*-Acetyl cysteine (NAC)	Accelerate wound healing	Accelerate wound healing	[109, 110]
